# The Chloroform Death-Bill for Twelve Months

**Published:** 1881-02

**Authors:** Ernest H. Jacob


### THE CHLOROFORM DEATH-BILL FOR TWELVE MONTHS.

BY ERNEST H. JACOB, M. D.

In a previous communication I reviewed the great change that had occurred during the past few years in the substitution of ether for chloroform in surgical practice. In 1876 a return from the anæsthetists of London and other large hospitals revealed the fact that, save for the aged and infants, ether had taken the place of chloroform in nearly all our great surgical centres, and not only this, but that the change was felt by the officers in charge of the administration to be one greatly to the advantage of the patients as regards their subsequent condition, as well as the mere avoidance of death while under the influence of the drug. And yet, during the past twelve months, no less than twenty-five deaths have occurred from chloroform—I ought rather to say, have been recorded, for, no doubt, many occur which it is convenient and possible to say nothing about. Of these twenty-five, seventeen only occurred in Great Britain, fifteen being in hospital, and eight in private practice. The operations were mostly of the simplest kind. Dressing wounded fingers or toes, three; necrosis, four; extraction of teeth, two; amputation of the breast and lithotrity, one each; and one for applying a splint to a child's leg. The deaths appear to have occurred in the usual way from sudden syncope. As regards the post-mortem appearances, which are noted in fourteen cases, in five all organs were perfectly healthy. In eight only the heart was more or less "fatty" or "flabby," and in one there was emphysema of the lungs. As regards locality, two deaths took place in the Edinburgh Infirmary, where, I believe, ether is not in use; two in the London Hospital, where ether is extensively used; and three in Liverpool. During this period five deaths from other anæsthetics have been recorded. One each from ethydene dichloride and ethyl dibromide, and three from ether. Of the latter two occurred in America, one during extraction of teeth, no particulars being given, and one during an operation for hip disease, the patient suffering from valvular disease of the heart. The remaining case, the only one in England, was that recently reported by Mr. Hartley, which could not be said to be due to the anæsthetic, as the patient was almost *in articulo* from intestinal obstruction when the operation was commenced. It can hardly be too strongly urged that the fatality of ananæsthetic cannot be deduced from the experience of one observer. The cases must amount to tens of thousands. During the past few years, however, there has been no lack of material for statistical inquiry, and the result is to be read in the facts I have stated. A death from an anæsthetic administered for a trivial operation cannot be regarded as anything but a serious calamity. It is not for me to say that a grave responsibility rests on the surgeon who employs chloroform when a safer anæsthetic is available, but I think it cannot be long before the voice of the profession (if not of the public, as in America) demands some explanation for the use of an agent whose victims are numbered by hundreds.—*London Lancet.*

